# Downregulation of Immunoglobulin-Like Transcript-4 (ILT4) in Patients with Psoriatic Arthritis

**DOI:** 10.1371/journal.pone.0092018

**Published:** 2014-03-27

**Authors:** Alberto Bergamini, Maria Sole Chimenti, Eleonora Baffari, Maria Domenica Guarino, Gianfranco Gigliucci, Carlo Perricone, Roberto Perricone

**Affiliations:** 1 Rheumatology, Allergology and Clinical Immunology, Department of Internal Medicine, Unit of Rheumatology, University of Rome Tor Vergata, Rome, Italy; 2 Reumatologia, Dipartimento di Medicina Interna e Specialità Mediche, Sapienza Università di Roma, Rome, Italy; University of London, St George's, United Kingdom

## Abstract

**Objective:**

The immunoglobulin-like transcript-4 (ILT4) is an inhibitory receptor that modulates the activity of innate immune agents. We determined the expression of ILT4 and analysed the relationship with the expression of costimulatory proteins and tumor necrosis factor-α (TNF-α) production in monocytes from patients with psoriatic arthritis (PsA) starting anti-TNF treatment.

**Methods:**

Peripheral blood monocytes from 15 healthy controls and from 16 patients with PsA were activated in vitro by CD40 ligand (CD40L) and analyzed for ILT4, CD40, CD80 and CD86 expression, and spontaneous lipopolysaccharide (LPS)-induced TNF-α production by flow cytometry, before and after treatment with adalimumab.

**Results:**

The percentage of ILT4-negative monocytes was greater in PsA patients compared to controls and negatively correlated with DAS44. Normal monocytes treated with sera of PsA patients showed a reduced expression of ILT4 compared with monocytes exposed to sera from controls. CD40, CD80 and CD86 expression was higher in patients compared to controls. Both spontaneous and LPS-induced TNF-α production was restricted to ILT4-negative monocytes and was greater in PsA patients compared to controls. Finally, twelve weeks-treatment with adalimumab resulted in a significant increase of ILT4 expression and a decrease of costimulatory molecules expression in PsA patients, compared to pre-therapy levels.

**Conclusions:**

These data support the possibility that changes in the immunophenotype of monocytes play a role in the pathogenesis of PSA. Thus, modulation of the expression of ILT4 may represent an enticing new therapeutic target.

## Introduction

Psoriatic arthritis (PsA) is a chronic inflammatory autoimmune disease that is characterized by inflammatory arthritis and psoriasis [1]. Patients frequently develop focal inflammation at multiple sites, including skin, nails, joints, and tendon-insertion sites or entheses [2]. Although there is still no direct evidence for the existence of arthritogenic peptides in PsA, current available data support the notion that T lymphocytes are important in the initiation and persistence of the chronic inflammatory process [3], [4]. However, more recent research has indicated that an exaggerated response of the innate immune system may also play an important role in the pathogenesis of this disease [5].

The interaction between the innate and adaptive immune systems is complex where among others, innate immunity play a pivotal role in directing aspects of adaptive immune responses. In this regard, a growing interest in the field of innate immunity has led to the identification of novel family of immune receptors known as immunoglobulin-like transcripts (ILTs), which recognise major histocompatibility complex (MHC) MHC class I. ILTs exert an immunomodulatory effect, which may be activating or inhibitory depending upon to the nature of intracellular signalling motifs and are expressed on a range of leukocytes including antigen presenting cells (APC) [6], [7]. Inhibitory ILT receptors have been shown to exert a negative influence on the stimulatory capacity of APC. High level of inhibitory ILT expression on the surface of APC inhibits NF-κB activation to prevent CD40-induced upregulation of costimulatory proteins [8], [9], with a consequent effect on T cells [10], [11]. Thus, inhibitory ILTs exert a direct influence on innate effectors or an indirect effect on an adaptive response and may play a potential role in PsA [12], [13]. Indeed, if inhibitory ILT signalling raises the activation threshold of T cells [14], their expression could act as an important autoregulatory mechanism in situations such as autoimmunity [15]. As opposed, lack of regulatory mechanisms in the joint could lead to inappropriate activity of innate immune agents, which may drive T-cell activation [16]. Indeed, silencing of inhibitory ILTs expression in APC has been found to increase T cell proliferation and synthesis of proinflammatory cytokines [17]. Although inhibitory ILTs appear to be upregulated in patients with rheumatoid arthritis [18], little is known about their expression in PsA.

In the present study, peripheral blood monocytes from patients with PsA were activated in vitro by CD40 ligand (CD40L), a molecule that plays a key role towards differentiation of these cells into APC [19]–[24], and then analyzed for the expression of the inhibitory receptor ILT4 [7], [25], costimulatory proteins (CD40, CD80 and CD86) and TNF-α production, before and after treatment with adalimumab.

## Materials and Methods

The study was carried out according to the Declaration of Helsinki and conducted in accordance with the International Conference on Harmonisation Good Clinical Practice Guidelines. The study protocol was approved by the ethic committee of the University of Rome Tor Vergata. All patients provided written informed consent before participating in any study-related activities.

### Patients and samples

The study included sixteen Caucasian patients 18 years or older, rheumatoid factor negative, with moderately to severely active PsA and had either active psoriatic skin lesions or a documented history of psoriasis, according to CASPAR criteria [26]. Patients were originally eligible for adalimumab if they presented unresponsiveness to at least two synthetic DMARDS or contraindications to conventional treatments including, methotrexate, cyclosporine, leflunomide, sulphasalazine [27]. Patients were naïve to biological agents. Fifteen race, age and sex matched healthy control subjects were recruited from a group of blood donors. Patients received subcutaneous injections of 40 mg adalimumab every other week. Blood samples were obtained just before the first administration of adalimumab (baseline) and at week 12 just before the seventh administration of adalimumab. Disease activity score (DAS) 44 was measured at baseline and at week 12, difference between DAS44 at baseline and week 12 are referred as ΔDAS44. This DAS is a continuous measure consisting of the variables Ritchie articular index, number of swollen joints, erythrocyte sedimentation rate and general health measured on a visual analogue scale [28]. Patients were recruited from the Rheumatology Unit of the University of Rome Tor Vergata.

### Compounds

Soluble trimeric CD40L was purchased from R&D system, San Diego CA. Lipopolysaccharide (LPS) from Escherichia coli 0111/B4 was purchased from Sigma Chemical Co. St. Louis, MO.

### Limulus amebocyte lysate test

All the compounds and media used in this study were analysed for endotoxin contamination by the limulus amebocyte lysate test (QCL-1000, BioWhittaker, Inc, Walkersville, MD). All the samples analysed were found free of endotoxin contamination (less than 0.1 EU/ml).

### Cell stimulation

Peripheral blood from controls or patients was enriched for peripheral blood mononuclear cells (PBMCs) by centrifugation over Ficoll Hypaque. PBMC were either analyzed by FACS for ILT4 expression on monocytes just after isolation or cultured in Roswell Park Memorial Institute (RPMI)-1640 medium supplemented with 20% heat-inactivated foetal calf serum (FCS), 2 mM L-glutamine, 50 U/ml penicillin, 50 μg/ml streptomycin and 500 ng/ml CD40L, referred to as complete medium. The cells were kept at 37°C in a humidified atmosphere of 5% CO_2_ in air, in 96 wells V bottom plates (Corning Incorporated, Corning, NY, catalogue number #3896) at a concentration of 5×10^5^ cells/well/250 μl for 72 h, in the presence or in the absence of 0.5 μg/ml CD40L and then analyzed by FACS for ILT4, CD40, CD80 and CD86 expression. To evaluate the ability of sera from PsA patients to modulate the expression of ILT4, monocytes were obtained from buffy coat preparation from healthy subjects by elutriation, as previously described [28]. Cells obtained by this method are >90% monocytes as determined by FACS analysis. Elutriated monocytes were dispensed at a concentration of 5×10^5^ cells/well/250 μl in complete medium without FCS, and cultured for 72 hours with the addition of either 20% sera from each of the PsA patients (obtained at baseline just before the first administration of adalimumab) and controls or 20% FCS (negative control), and then analyzed by FACS for ILT4 expression. To evaluate TNF-α at the end of the incubation period the cells were washed, and fed with fresh complete medium containing 1 μg/ml of the protein transport inhibitor brefeldin A (Sigma Chimica, Milan, Italy), with or without 100 ng/ml LPS. Sixteen hours after brefeldin A addition the cells were analysed by FACS for intracellular cytokine production.

### Flow cytometry studies

For fluorescence-activated cell sorting (FACS), cells were washed once with washing buffer (3% (v/v) FCS and 0.1% (w/v) NaN_3_ in PBS). 2×10^5^ cells were incubated with the following antibodies at appropriate dilutions for 30 min at 4°C in the dark: R-phycoerythrin-cyanine (PC5)-conjugated anti-CD14 (Immunotech), phycoerythrin (PE)-conjugated anti- ILT4 (Immunotech, Marseille, France, clone: PN A22334), fluorescein isothiocyanate (FITC)-conjugated anti-CD40, anti-CD80, anti CD86 (BD Biosciences San Josè CA), and anti-TNF-α (R&D system, San Diego CA). For the determination of intracellular cytokine production, at the end of the incubation period, the cells were washed with washing buffer and stained with anti-CD14 and anti-ILT4 as described above. Cells were then permeabilized in Cytofix/Cytoperm solution (Pharmingen), stained with anti-TNF-α or anti-IL-1β for 30 min at room temperature, then analysed by FACS. The negative range and staining specificity for each cell surface marker and cytokine was established using a corresponding isotype-matched control antibody, conjugated with the same fluorescent dye. Flow cytometry was performed using a FACS can flow cytometer and analyzed with Cell Quest software (Beckton Dickinson). For each analysis, 10^4^ events were gated on CD14 expression and a light scatter gate designed to either include only viable cells (surface markers determination) or exclude cellular debris (determination of intracellular cytokine production) [29].

### Statistics

The normality of variable distribution was assessed by the Kolmogorov-Smirnov goodness of fit test. Comparisons between distributions of two variables for a single group were performed by Student's unpaired t test or Mann Whitney U test, and presented as mean ± standard deviation (SD) or median with 25th and 75th percentile where appropriate. Correlations were evaluated by the Pearson parametric test for the univariate analysis. All *P* values are 2-tailed. *P* values <0.05 were considered significant. All statistical analyses were performed using commercial available statistical package (SPSS for Windows, Version 10.0).

## Results

### Patients characteristics

The demographic and clinical characteristics of patients and controls are given in Table 1. Out of the sixteen patients, twelve were moderate to good responders, defined as per the EULAR criteria with a change higher than 1.2 of the DAS44 at week 12 with respect to baseline, and 4 were non-responders. The mean DAS44 at week 12 improved by 68.2% (mean ΔDAS44 2.51±1.81).

**Table 1:** Demographic and clinical characteristics of the patients and controls at baseline.

	Patients	Controls	
Age (years, mean±SD)	47.3±11.5	44.7±15.7	*P* = 0.64
Sex, No. male/female	5/11	6/9	*P* = 0.72
Disease duration (mean±SD)	6.3±7.3	n.a.	
DAS44 (median and interquartile range)	4.3 (3.4–4.9)	n.a	
CRP (mg/liter, median and interquartile range)	4.7 (0.1–13.5)	<0.1	
ESR (mm/hour, median and interquartile range)	42±27.6	<20	
**Medications**			
NSAID (No./%)	3 (18.8)	n.a	
Corticosteroids (No./%)	7 (43.8)	n.a	
DMARD (No./%)	11 (69.8)	n.a	

n.a  =  not applicable; DAS  =  disease activity score; CRP  =  C-reactive protein; ESR  =  erythrocyte sedimentation rate; NSAID  =  non-steroidal anti-inflammatory drugs; DMARD  =  disease-modifying antirheumatic drugs.

### ILT4 expression on CD40L-monocytes of patients and controls

**Figure 1:**
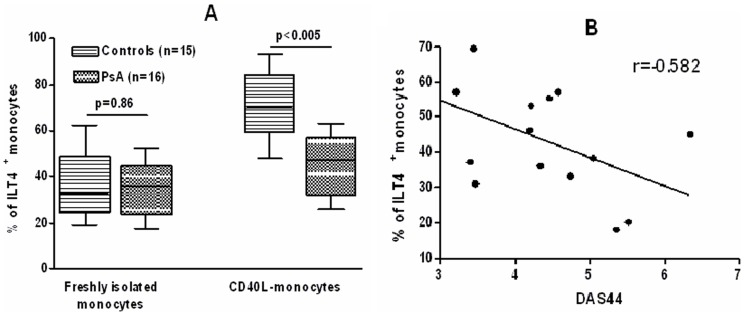
Expression of ILT4 on monocytes from controls and patients with PsA before and after *in vitro* treatment with CD40L. (**A**) Cells were gated for monocytes based on their forward-scatter/side-scatter profile. Controls with isotype matched irrelevant mAbs consistently showed <1% of positive cells. The data represent mean±SD (error bars). MFI values of ILT4 expression in CD40L-stimulated cells were as follows. Freshly isolated monocytes: controls, 44.6±9.8, PsA, 40±12.3, *P*>0.05. CD40L-monocytes: controls, 97.5±29.8, PsA, 57.1±15, *P*<0.005. Comparison between means was performed by Student's unpaired t test. All *P* values are 2-tailed. *P* values <0.05 were considered significant. (**B**) Correlation between monocyte ILT4 expression and DAS44. ILT4 expression negatively correlated to DAS44 (*P* = 0.02). Correlations were evaluated by the Pearson parametric for the univariate analysis.

**Figure 2:**
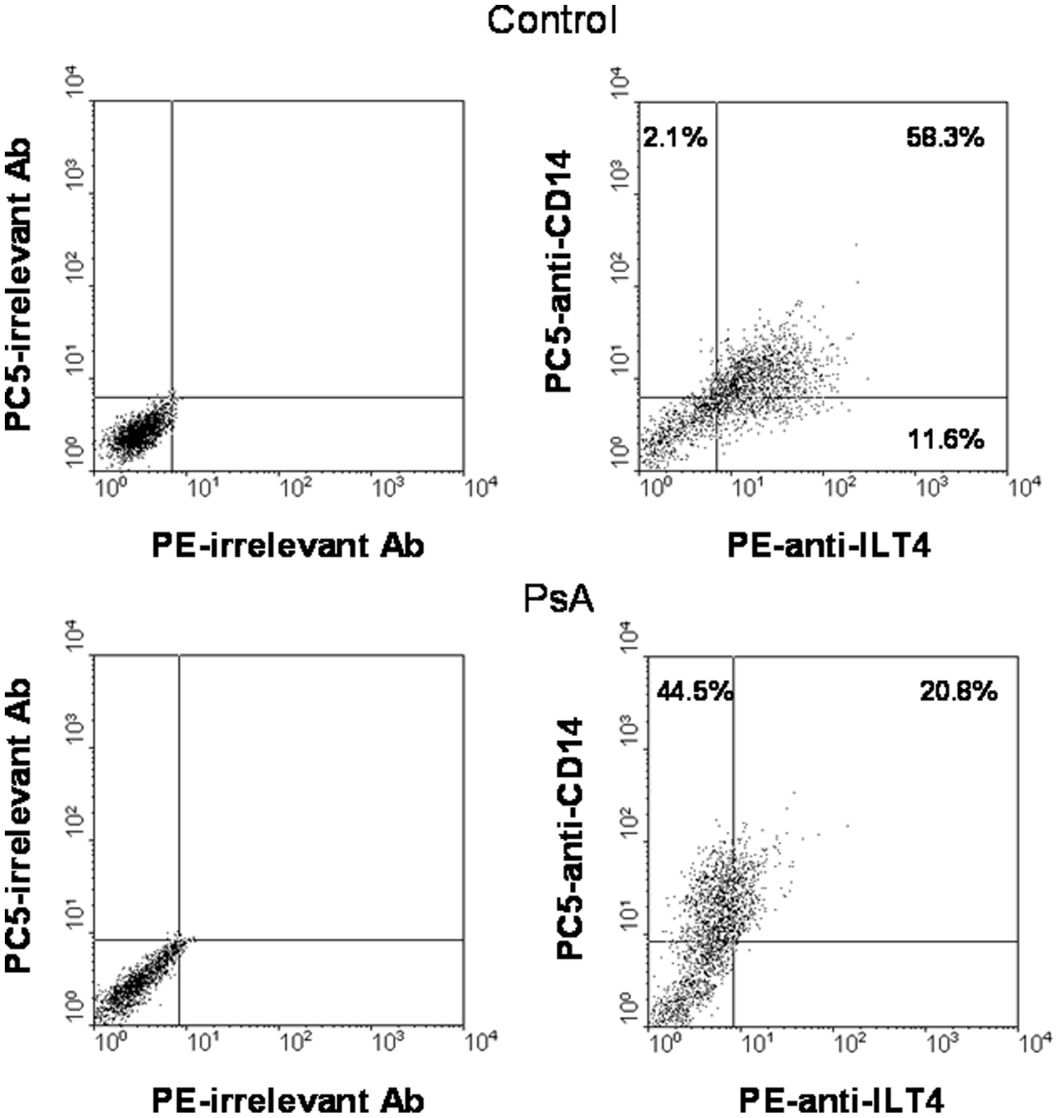
Measurement of ILT4 expression in a representative control subject and a patient with PsA. Dot plots showing CD14/ILT4 co-expression in CD40L monocytes from representative control and PsA subjects.

**Figure 3:**
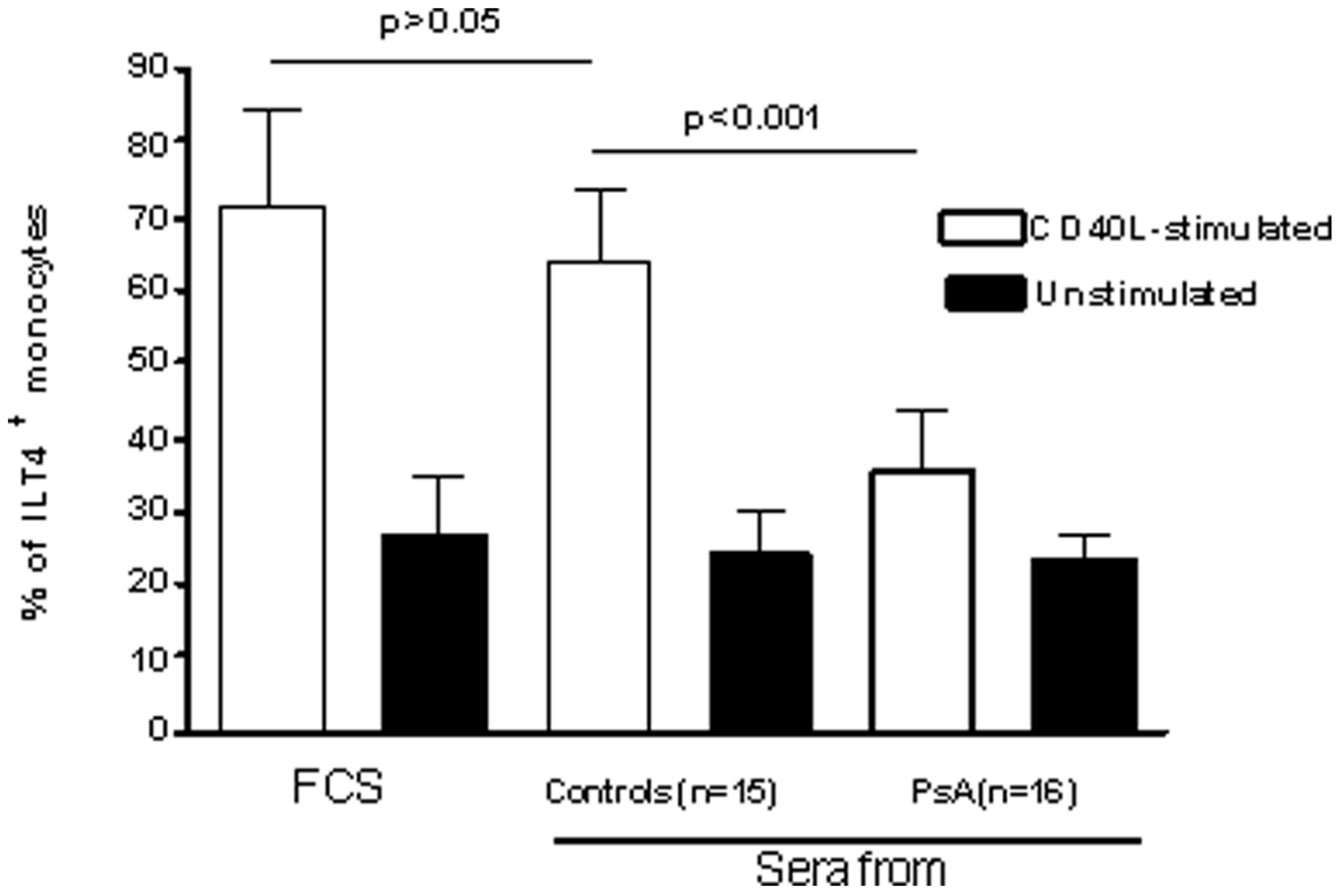
Sera from PsA patients downmodulate ILT4 expression in CD40L-stimulated, normal monocytes. Sera from each of the 16 patients with PsA and of the 15 controls were incubated with elutriated monocytes from a single healthy donor. Sera from patients and controls were obtained at baseline. Medications of patients at baseline are reported in Table 1. FCS was used as negative control. The experiments were performed twice, using cells from two different healthy donors; each experiment was carried out in triplicate wells. The data represent mean±SD (error bars). Comparison between means was performed by Student's unpaired t test. MFI values of ILT4 expression in CD40L-stimulated and, in parenthesis, unstimulated cells were as follows: FCS-exposed, 145±13.9 (34.5±12.4); normal serum-exposed, 120.3±11.2 (30.9±13.7); patient's serum-exposed 33.6±8.4 (36.1±14.6). CD40L: FCS-exposed vs. normal serum-exposed, *P*>0.05; normal serum-exposed vs. patient's serum-exposed, *P*<0.001. Unstimulated: FCS-exposed vs. normal serum-exposed, and normal serum-exposed vs. patient's serum-exposed, *P*>0.05.

Data on the surface expression of ILT4 on freshly isolated monocytes and monocytes cultured for 72 h in the presence of CD40L CD40L are summarized in Fig. 1A. The percentage of freshly isolated monocytes that expressed ILT4 was similar in patients and controls. After 72 h of culture in the presence of CD40L, the percentage of monocytes from normal subjects that expressed ILT4 increased by about two fold as compared to freshly isolated monocytes. In contrast, the percentage of patient's monocytes that expressed ILT4 was similar to that found in freshly isolated cells. Pearson's correlation analysis showed that the expression of ILT4 on CD40L-monocytes from patients negatively correlated with DAS44 (r = −0.582, confidence interval = −0.836 to −0.121, *P* = 0.02) (Fig. 2B). Fig. 2 shows dot plots of ILT4 expression on monocytes from a representative control subject and a patient with PsA. Then, sera from patients and controls were tested for the ability to modulate ILT4 expression in purified monocytes from normal donors, treated or not with CD40L (Fig.3). CD40L over modulated the expression ILT4 as compared to unstimulated cells. A significant reduction of the expression of ILT4 was observed in monocytes from normal donors stimulated by CD40L, No differences in the expression of ILT4 were detected in unstimulated cells. In the presence of individual specimens of sera of patients compared to monocytes incubated in the presence of sera of control subjects.

### CD40, CD80 and CD86 expression on CD40L-monocytes of patients and controls

**Figure 4:**
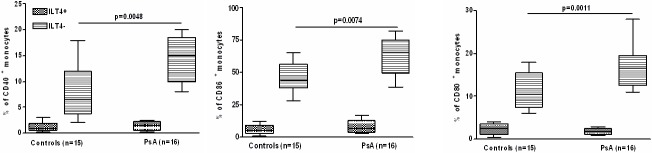
Expression of costimulatory molecules on ILT4^+^ and ILT4^−^, CD40L-stimulated monocyte subsets. The box plots represent the expression of ILT4 analyzed by FACS on CD14^+^ gated cells as percentage of positive cells. Controls with isotype matched irrelevant mAbs consistently showed <1% of positive cells. The data represent means±SD (error bars) Comparison between means was performed by Student's unpaired t test.

CD40L-monocytes were analyzed with regard to the expression of the costimulatory molecules CD40, CD80 and CD86 (Fig.4). CD40 CD80 and CD86 were mostly expressed on ILT4^−^ monocytes and their expression was significantly higher in cells from patients compared to controls. In contrast, only a small percentage of ILT4^+^ monocytes were positive for CD40, CD80 and CD86, and their expression was not different between patients and controls.

### TNF-α production in monocytes from patients and controls

**Figure 5:**
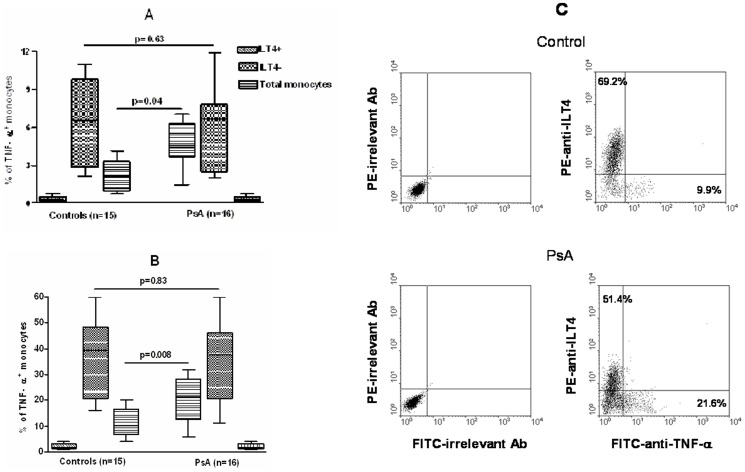
Spontaneous and LPS-induced TNF-α production by total monocyte population and ILT4^+^ and ILT4^−^ monocyte subsets from patients and controls. The box plots represent the spontaneous (A) and LPS-induced (B) intracellular production of TNF-α analyzed by FACS as percentage of positive cells. Appropriate controls with isotype matched irrelevant mAbs were carried out and consistently showed <1% of positive cells. The data represent means±SD (error bars). Comparison between means was performed by Student's unpaired t test. (C) LPS-induced TNF-α production by total monocyte population from a representative control subject and a patient with PsA. CD40L-activated monocytes were stimulated with LPS and stained with PE-anti-ILT4 for the determination of surface phenotype. Intracellular TNF-α was detected by staining with FITC-anti-TNF-α. The data are displayed as dot plots.

Spontaneous and LPS-induced TNF-α production was analyzed at single cell level in total monocyte population and ILT4^+^ and ILT4^−^ monocyte subsets from patients and controls (Fig.5, A and B). Analysis of monocyte subsets revealed that both spontaneous and LPS-induced TNF-α production was restricted to ILT4^−^ monocytes. No difference in TNF-α production was observed in ILT4^−^ monocyte subset from patients and controls. Nonetheless, in total monocyte population the percentage of cells positive for intracellular TNF-α was greater in patients compared to controls because of the difference in the percentage of ILT4^−^ cells between the two groups. Fig. 5C shows FACS plots of LPS-induced TNF-α production by total monocyte population from a representative control subject and a patient with PsA.

#### Effect of adalimumab treatment on the surface and cytokinic phenotype of monocytes

**Figure 6:**
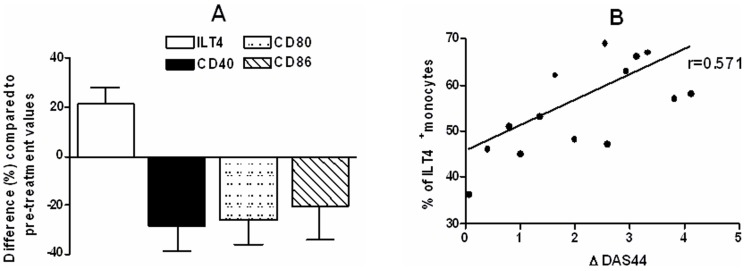
Effect of anti-TNF-α therapy on surface phenotype of CD40L-activated total monocytes in patients with PsA. (**A**) Median and interquartile range of percentage of monocyte expressing ILT4, CD40, CD80 and CD86 from sixteen patients at baseline vs. week 12 were as follows: ILT4, 45.5 (34–57) vs. 57 (47.5–65.5), P = 0.016; CD40, 15 (10–18.5) vs. 11 (7–12.5), P = 0.025; CD80, 16.6 (12.5–19.5) vs. 12 (8–15), P = 0.015; CD86, 65.5 (49.5–75) vs. 49.5 (41.5–61.5), P = 0.036. Comparison between means was performed by Student's unpaired t test. (**B**) Correlation between monocyte ILT4 expression and ΔDAS44 at week 12 after adalimumab. ILT4 expression directly correlates to ΔDAS44 (*P* = 0.02) Correlations were evaluated by the Pearson parametric for the univariate analysis.

Analysis of ILT4 and costimulatory molecules expression in CD40L-activated monocytes of patients with PsA before and after treatment with adalimumab revealed a significant increase of ILT4 expression and a decrease of costimulatory molecules expression, compared to baseline levels (Fig.6A). Moreover, a positive correlation was found between the level of ILT4 expression at week 12 and ΔDAS44 (r = 0.571, confidence interval = −0.103 to −0.83, *P* = 0.02) (Fig 6B). No effect on ILT4 and costimulatory molecules expression was observed in normal CD40L-activated monocytes treated in vitro with concentrations of adalimumab as high as 10 μg/ml (data not shown).

## Discussion

Our results showed a similar expression of ILT4 in PsA patients and controls on circulating monocytes. In previous studies, ILT4 expression was found decreased on circulating monocytes of patients with juvenile idiopathic arthritis (JIA) [30], whereas, an abundant expression of ILT4 was detected on macrophages in the synovium of patients with RA [18]. These results imply that the pathogenesis of these disease is heterogeneous, in particular in the mechanisms of regulation and control of the conjugation process of the T-cell and the APC in the formation of the immunologic synapse.

At variance with peripheral blood monocytes, that showed no significant differences in ILT4 expression between patients and controls, monocytes of patients with PsA when cultured in the presence of CD40L expressed lower levels of ILT4 compared with cell from controls. The work of Suciu-Foca and co-workers has demonstrated the potent tolerogenic effects of ILT4 [31]. APC expressing high levels of ILT4 anergize allospecific CD4^+^ CD45RO^+^ CD25^+^ T cells and convert them into regulatory T (Treg) cells, propagating further the T-cell suppression cascade [31]. The regulatory properties of ILT4 are mediated by a failure to upregulate the costimulatory proteins CD40, CD80 and CD86, hindering their ability to activate CD4^+^ helper T (Th) cells [8], [32]. Indeed, we found that CD40, CD80 and CD86 were mostly expressed on ILT4^−^ monocytes and their expression was significantly higher in cells from patients compared to those from controls. Thus, the downregulation of ILT4 expression in addition to upregulation of the costimulatory molecules suggests that monocytes of patients with PsA acquire an activated/non-tolerogenic phenotype. This activated/non-tolerogenic phenotype might contribute to abnormally high APC activation, which is likely to significantly affect the development of T cell mediated immune response. When debating the potential importance of innate versus adaptive immune systems in autoimmune diseases, it is essential to consider the interdependence of the two systems. Other immune agents may in fact mediate the pathogenic phase of a T cell dependent disease. T lymphocytes play a pivotal role in the pathogenesis of PsA. CD4^+^ and CD8^+^ T cell subsets of memory phenotype are present in large numbers in the PsA synovia. Studies examining clonality, usually employing T cell receptor (TCR) immunospectrotyping analyses, suggest that local antigen recognition may drive at least a subset of these T cells, although few autoimmune targets have been identified in PsA, perhaps reflecting the absence thus far of defined autoantibodies [3], [4]. Without the influence of inhibitory receptor signalling, the threshold for APC activation is lowered. Inappropriate activity of APC in the affected joints together with the accumulation of activated T-cells may drive the initiation and persistence of the T-cell activation. Consistently, the role of CD28/CD80 in experimental models of psoriasis and in humans has been recently reviewed [33]. It was concluded that CD28/CD80 is crucial in promoting T cell inflammation in psoriasis and the effect of blocking CD28/CD80 signalling by CTLA-4 analogues or by anti-CD28 blocking antibodies is effective against PsA. In line with the possible presence of a dysregulation of innate immune receptors for self in PsA, low plasma levels of soluble HLA-G, the high-affinity ligand for ILT4, were found in patients with psoriasis compared to healthy controls [34].

As well as being involved in functional shaping of T cell responses toward a tolerant state, ILT4 also plays a critical role in control of inflammation by modulating the proinflammatory cytokine secretion profile [35], [36]. Here, we found that ILT4^−^ cells were proficient at producing TNF-α while no production of this cytokine could be detected in ILT4^+^ cells. In particular, ILT4^+^ monocytes failed to produce TNF-α without actively ligating ILT4 on their cell surface. Indeed, ligation or cross-linking of inhibitory ILT receptors results in tyrosine phosphorylation of receptors and recruitment of the SH2-containing tyrosine phosphatase SHP-1, which are involved in regulation of cytokine production and the cytokine-receptor signaling pathway [37]. However, mRNA analysis in ILT4^+^ dendritic cells (DC), showed inhibited mRNA expression of relB [35], in the absence of ligation or cross-linking of ILT4. RelB is a NF-kB transcription factor, and its nuclear expression is related to DC maturation [7]. Interestingly, both the inflammatory cytokine IL-12p40 mRNA expression and production of IL-12 protein were also significantly lower [35], [36]. Thus, it is possible that the inhibition of relB expression may contribute to the arrest of ILT4^+^ CD40L-monocytes at an immature state with reduced ability to produce TNF-α[. Although we did not detect significant difference in the cytokine profile of ILT4^−^ cells between patients and controls, the increased percentage of ILT4^−^ monocytes in patients vs. controls resulted in greater TNF-α production by total monocyte population from patients compared with controls. TNF-α is a key proinflammatory cytokine with an important pathogenic role in the chronic inflammation found in PsA patients. The distribution of TNF-α expression in PsA is similar to that described in rheumatoid arthritis although this cytokine levels in the psoriatic form may be somewhat greater than in RA [38], [39]. TNF-α is produced by monocyte/macrophages and has been found localized to perivascular macrophages in the PsA synovia [40]. Thus, it is possible that downregulation of ILT4 can cause excess inflammation in the affected joints, leading to destruction of tissue. Interestingly, Chang CC et al., [17] reporting on ILT3, another member of the family of inhibitory ILTs, have shown that ILT3-silenced-DC, other than augment proliferation of T cells, induced overactive inflammation accompanied by a more vigorous proinflammatory cytokine response when compared with DC which express physiological levels of ILT3 in response to “danger” signals relayed through a variety of toll like receptors (TLR). TLR are a type of pattern recognition receptors that recognize molecules that are broadly shared by pathogens but distinguishable from host molecules [41], and it has been suggested that they may be involved in PsA, in the recognition of microbial products. This may be important since previous studies have demonstrated the presence of a wide variety of bacterial species in the synovia of patients with PsA [42]–[44].

The interaction between CD40L and its receptor CD40 on monocytes is an essential step for triggering the adaptive immune response [20]–[24]. CD40-CD40L interactions are also thought to regulate a number of functions that may be important in PsA pathogenesis, such as increased IL-12 production, a key-inducer of Th1 responses by antigen presenting cells [45], [46], the induction of proinflammatory cytokines and the up-regulation of adhesion molecules [47], [48]. In addition, CD40L is overexpressed on T-cells from patients with active PsA, possibly indicating a role for CD40L in PsA pathogenesis [49].

Upregulation of ILT4 in monocytes occurs upon interaction with antigen-specific CD8^+^ CD28^−^ suppressor cell, or upon exposure to inhibitory cytokines [8]. Consistent with previously published data from our laboratory [50], we show here that CD40L upregulated ILT4 expression on normal monocytes. We also show that that sera from patients with PsA downregulated ILT4 expression in CD40L-activated normal monocytes. This may be of interest because blood monocytes migrate to the joints to become synovial macrophages. The possibility that the serum of patients with PsA may have a direct effect on ILT4 expression seems unlikely because no modulation of ILT4 expression was observed in the absence of CD40L-activation, moreover, the expression of ILT4 in circulating monocytes of patients with PsA was found not dissimilar to that observed in normal subjects. From these observations it is possible to speculate that one or more substances contained in the serum of patients with PsA modulate defined intracellular pathways that interfere with the effects of CD40L on monocyte differentiation. Following this line of reasoning, Lehtonen et al. found that the transcription factor, IFN regulatory factor 4 (IRF4) is induced during monocyte differentiation [51]. In addition, the work of Nakajima et al. has demonstrated that the transcription factor PU.1 regulates ILT4 expression and the observation that IRF4 binds to PU.1 highlights a potential mechanism for the regulation of ILT4 during monocyte maturation [52]. It may be proposed that the reduction in PU.1 as a result of its interaction with IRF4 contributes to the downregulation of ILT4 expression during the course of monocyte differentiation as reported in our study.

A significant increase of ILT4 expression and a decrease of costimulatory molecules expression were documented in monocytes from PsA patients after 12 weeks of treatment with adalimumab. The increase of ILT4 expression in patients after treatment could be related to the ability of adalimumab to block in vivo the effects of TNF-α, which may act in reducing ILT4 expression on APC. However, the direct relation found between the level of ILT4 expression after treatment and ΔDAS44 suggests that the effect of adalimumab on monocyte surface phenotype may be due to the modification of the inflammatory milieu associated with therapy-induced reduction of disease activity.

Some limitations of this study deserve consideration. First, bystander activation of other leukocytes in the PBMC is a theoretically possible mechanism for inflating the number of responding cells in bulk culture experimental systems. This would not be expected with Brefeldin A-treated cultures and flow cytometric assay. Brefeldin A is a relatively non-toxic, but potent, inhibitor of cellular transport that efficiently prevents potentially stimulatory cytokines or cell surface adhesion molecules from being secreted or transported to the cell surface in these cultures [53]. Second, we analyzed CD40L-activated monocytes and not dendritic cells. Although CD40L has been shown to promote the differentiation of blood monocytes into functional DCs, in the absence of other activating factors, monocyte maturation into dendritic cells by CD40L alone requires a stimulation period of seven days, which is quite longer than that used here [24]. Moreover, we did not check for the presence of dendritic cells in our CD40L-stimulated cultures. Third, cell samples and the sera used in this study were obtained from patients who were on corticosteroids and/or disease-modifying antirheumatic drug (DMARD). These drugs have the potential to modulate the phenotype of monocytes. Indeed, several inhibitory effects of corticosteroids and methotrexate on monocyte functions have been reported *in vitro* and *in vivo* [54], [55].

In conclusion, our data support the possibility that changes in the immunophenotype of monocytes play a role in the pathogenesis of PSA. In line, modulation of the expression of ILT4 may represent an enticing new therapeutic target.
